# Impact of lipopolysaccharides on cultivation and recombinant protein expression in human embryonal kidney (HEK‐293) cells

**DOI:** 10.1002/elsc.202100065

**Published:** 2021-10-25

**Authors:** Christine Faust, Christian Beil, Werner Dittrich, Ercole Rao, Thomas Langer

**Affiliations:** ^1^ R&D Biologics Research, Building H811 Sanofi‐Aventis Deutschland GmbH Frankfurt am Main Germany

**Keywords:** endotoxin, HEK‐293 cells, lipopolysaccharides, recombinant protein expression, transient transfection

## Abstract

The human embryonal kidney 293 cell (HEK‐293) is a widely used expression host for transient gene expression. The genes or plasmids used for the transient transfections are usually propagated and extracted from the gram‐negative bacterium *Escherichia coli*, the workhorse for molecular biologists. As a gram‐negative bacterium *E. coli* has an outer membrane (OM) containing lipopolysaccharides (LPS) or endotoxins. LPS are very potent inducers of inflammatory cytokines in the body. In early research phases DNA intended for transient transfections is not routinely checked for LPS‐levels. In this study we addressed the question whether LPS has an impact on the cultivation and production of a recombinant antibody. At high concentrations the presence of LPS has a detrimental impact on cell viability and recombinant protein expression. But low LPS concentrations are tolerated and might even enhance protein expression levels.

**Abbreviations**
EU: endotoxin units
LPS: lipopolysaccharide
MD2: myeloid differentiation factor 2
TLR4: toll‐like receptor 4
UPR: unfolded protein response

## 1 INTRODUCTION

The market for biopharmaceuticals has increased significantly within the recent years. Biopharmaceuticals comprise recombinant produced proteins like monoclonal antibodies, peptide hormones, or Fc‐fusion proteins (peptidbodies; biologic active peptides fused to the fragment crystallizable [Fc] domain of an immunoglobulin G). So far, more than 1200 protein therapeutics have been approved by the FDA as listed in the THPdb [1]. Most of the therapeutic used proteins and peptides are made in a recombinational manner that is being produced by living cells. The most frequently used expression systems are either microbial (bacteria and yeast) or based on mammalian cell systems. Production of peptides and small proteins is usually done in microbial systems for financial reasons whereas larger and more complex proteins, for example, monoclonal antibodies, are produced in mammalian cells [2]. The most prevalent used mammalian expression systems are Chinese hamster ovary (CHO) and human embryo kidney 293 (HEK‐293) cells. CHO cells are the preferred host for large scale production whereas HEK‐293 cells are the preferred expression system for small scale expression in research due its high transfection efficiency and ease of cultivation [3, 4]. During the discovery phase for novel protein therapeutics or antibodies hundreds or thousands of different protein constructs are produced in transiently transfected HEK‐293 cells and screened for desired properties [5]. The gene of interest is usually placed under a strong promotor, for example, the viral SV40 or cytomegalovirus (CMV) promotor in a DNA plasmid. Transient transfection of HEK‐293 cells is usually done by mixing the DNA with polyethylenimine and incubating the cells with the transfection mixture [6, 7]. After transfection, cells are further cultivated for several days to allow for protein expression. The DNA used for mammalian cell transfection is usually cloned, propagated and purified from the bacterium *Escherichia coli*.

**PRACTICAL APPLICATION**

Lipopolysaccharids (LPS) are very potent immune stimulators for eukaryotic cells and at certain concentrations they are toxic. The HEK293 cell line is the working horse for recombinant protein production in biopharmaceutical research for protein production. The DNA used for transfection is obtained from *E. coli* and might still contain residual levels of LPS. A recurring question is whether contaminating LPS levels have an impact on transient protein expression. In our article the impact of LPS into the culture medium on HEK293 growth and transient protein expression was systematically investigated. We not only calculated the LPS concentrations but also measured the LPS levels after addition to the medium which was not done in earlier publications. The main outcome of our study is that low level of LPS are not harmful and might even enhance protein expression.

*E. coli* is a gram‐negative bacterium and as such it is enveloped with two membranes: an inner membrane (IM) surrounding the cytoplasm and an outer membrane (OM) shielding the bacterial cell from the environment. Between the two membranes is the so‐called periplasmic space, containing the peptidoglycan bacterial cell wall. In contrast to the IM, which is a phospholipid bilayer, the OM has a unique architecture. The OM is an asymmetric bilayer containing lipopolysaccharides (LPS) in the outer leaflet [8]. LPS are unique for gram‐negative bacteria and most animals are able to respond to very small amounts of LPS. Since LPS are not made by animals, the presence of LPS is an indication of infection. Indeed, LPS are probably the most potent activators of the human immune system [9].

The DNA used for transient transfection is made from *E. coli* and usually not specifically tested for LPS‐content. Hence, it cannot strictly be ensured that the DNA used for transient transfection is indeed completely free from LPS. In our study we investigated the impact of different amounts of LPS added to transiently transfected HEK‐293 cells on cell proliferation and protein expression. We chose adalimumab as model protein since this monoclonal antibody is usually expressed in high yields.

## 2 MATERIALS AND METHODS

### 2.1 Antibody construct and cell cultivation

The nucleotide sequences coding for the light chain and heavy chain of adalimumab (Drug Bank access: DB00051) were synthesized (Thermo Fisher Scientific) and each cloned into an expression vector under a CMV promoter. A leader sequence was added to direct protein expression into the culture supernatant. Human embryo kidney cells (Freestyle 293 F, Gibco) were cultivated in non‐baffled shake flasks (Corning) 110 rpm, 37°C and 8% CO_2_ in FreeStyle F17 medium (Gibco) supplemented with 6 mM glutamine (Gibco). A culture at 1 L scale was grown to a cell density of 1.5 × 10^6^ cells/mL and split into two subcultures. One subculture was transfected and the other remained non‐transfected. Plasmids coding for the heavy and light chains were used in a 1:1 stoichiometry and mixed with linear polyethyleneimine (PEI) in a ratio of 1:3 in Opti‐MEM‐I medium (Gibco). After incubation for 20 min the transfection mixture was added to the cell culture. Subsequently, both cultures were further divided into 50 mL cultures in 250 mL non‐baffled shake flask (Corning) and different amount of LPS were added. LPS was either dissolved directly to the cell culture or added from a 1 mg/mL stock solution made with FreeStyle F17 medium. LPS was from *Escherichia coli* strain O111:B4 (Sigma Aldrich, #L2630). As stated by the manufacturer, the LPS preparation contains endotoxin levels of not less than 500,000 EU/mg. The calculated target LPS levels are based on the relation that 1 ng of LPS is equivalent to 0.5 endotoxin units (EU). After addition of LPS, cell cultures were cultivated for further 7 days.

### 2.2 Cell counting and antibody‐titer determination

During continued cultivation aliquots were withdrawn at different time points for cell counting and antibody titer determination. Determination of cell number, cell diameter and percentage of cell aggregation were done using an automated cell counter (Nucleocounter NC‐200, Chemometec, Allerod, Denmark) in the Via1‐Cassette (Chemometec). Data were analysed with the NucleoView NC‐200 software (Chemometec). For antibody titer determination an aliquot of the cell culture was centrifuged (13.000 rpm, 5 min) and the supernatant was analysed using a BLItz device (Fortebio) equipped with Protein A biosensors (Fortebio) for 60 s and 2200 rpm shaker speed. Antibody concentrations were determined using a corresponding calibration curve as described in the manufacturer´s manual.

### 2.3 LPS‐determination

LPS concentrations in the cell culture supernatants were determined using the Endosafe PTS device or the Endosafe nexgen‐MCS device (Charles River). The assay principle is based on the Limulus amebocyte lysate (LAL) assay. Used cartridges had a detection sensitivity of 10–0.1 EU/mL (#PTS20F, Charles River) and supernatants were diluted accordingly before measurements with sterile H_2_O. Measurements were done following the manufacturers instruction. Results were given in EU/mL.

### 2.4 SDS‐PAGE

Samples from supernatants were checked at day 6 by SDS‐PAGE under either non‐reducing or reducing conditions. LDS‐sample buffer (Invitrogen) was used as loading buffer. For samples analysed under reducing conditions, 0.1 M DTT was added to the sample buffer and samples were incubated for 5 min at 99°C. Samples were loaded on a 4%–12% bis‐tris gel (NuPAGE gel with Mes‐running buffer, Invitrogen) and run for 40 min at constant 200 V. The size marker used was the BenchMark protein ladder (Invitrogen). Gels were stained with coomassie‐blue (Instant blue, Expedeon, Harston, Cambridgeshire, UK) and destained with water.

## 3 RESULTS

**FIGURE 1:**
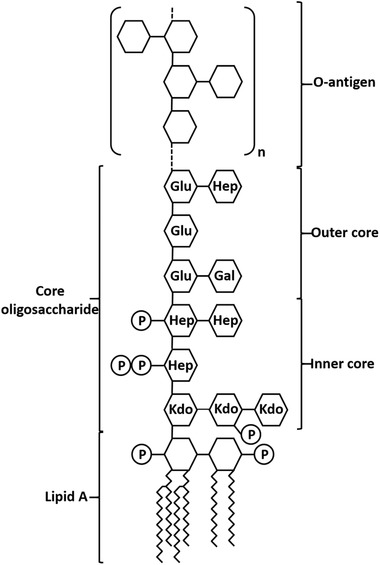
General structure of LPS from E. coli. The lipid A part is composed of a di‐glucosamide backbone with two phosphoryl groups and up to four fatty acid chains. It is the lipid A part of LPS which is responsible for the endotoxic activity. The carbohydrate part is divided into a core polysaccharide part and a so‐called O‐antigen. The core polysaccharide consists of a conserved inner core containing unusual sugars, mainly 2 keto‐3‐deoxyoctonate (Kdo) and heptose (Hep). The outer core is made of common hexoses such as glucose (Glu) and galactose (Gal). The core oligosaccharide can be further modified, for example, phosphorylated. The O‐antigen is a polysaccharide composed of a variable number of repeating oligosaccharide building blocks and is in general highly diverse

LPS are unique for gram‐negative bacteria. DNA used for transient transfection is mostly purified from the gram‐negative bacterium *E. coli* and might hence be contaminated with LPS. In this study LPS from *E. coli* (Figure 1) was added to HEK‐293 cell cultures to evaluate the impact on cell viability and recombinant protein production. The LPS used was a commercial available LPS preparation from the *E. coli* strain O111:B4, which is frequently used to stimulate immune response [10, 11]. This strain differs in LPS composition certainly from those *E. coli* strains used for plasmid preparation. The differences between LPS structures form diverse *E. coli* species are mainly within the O‐polysaccharide whereas the lipid A portion is built by a conserved biochemical pathway [12, 13]. It is the lipid A component of LPS that binds to and activates the TLR4/MD2 receptor complex [14, 15]. Hence, it can be assumed that the ability of LPS preparations from different *E. coli* species to evoke TLR4 signalling is similar.

Three completely independent experiments were performed. The starting cell cultures are derived from continuously cultivated cell cultures and, since the three experiments were conducted at different times points, the starting cultures had different number of passages after recovery from the master stock stored in liquid nitrogen. Starting culture for experiment 1 was passage P32. For experiments 2 and 3 starting cultures were derived from a different recovery from the cryo‐stock and were passages P10 and P30, respectively.

**FIGURE 2:**
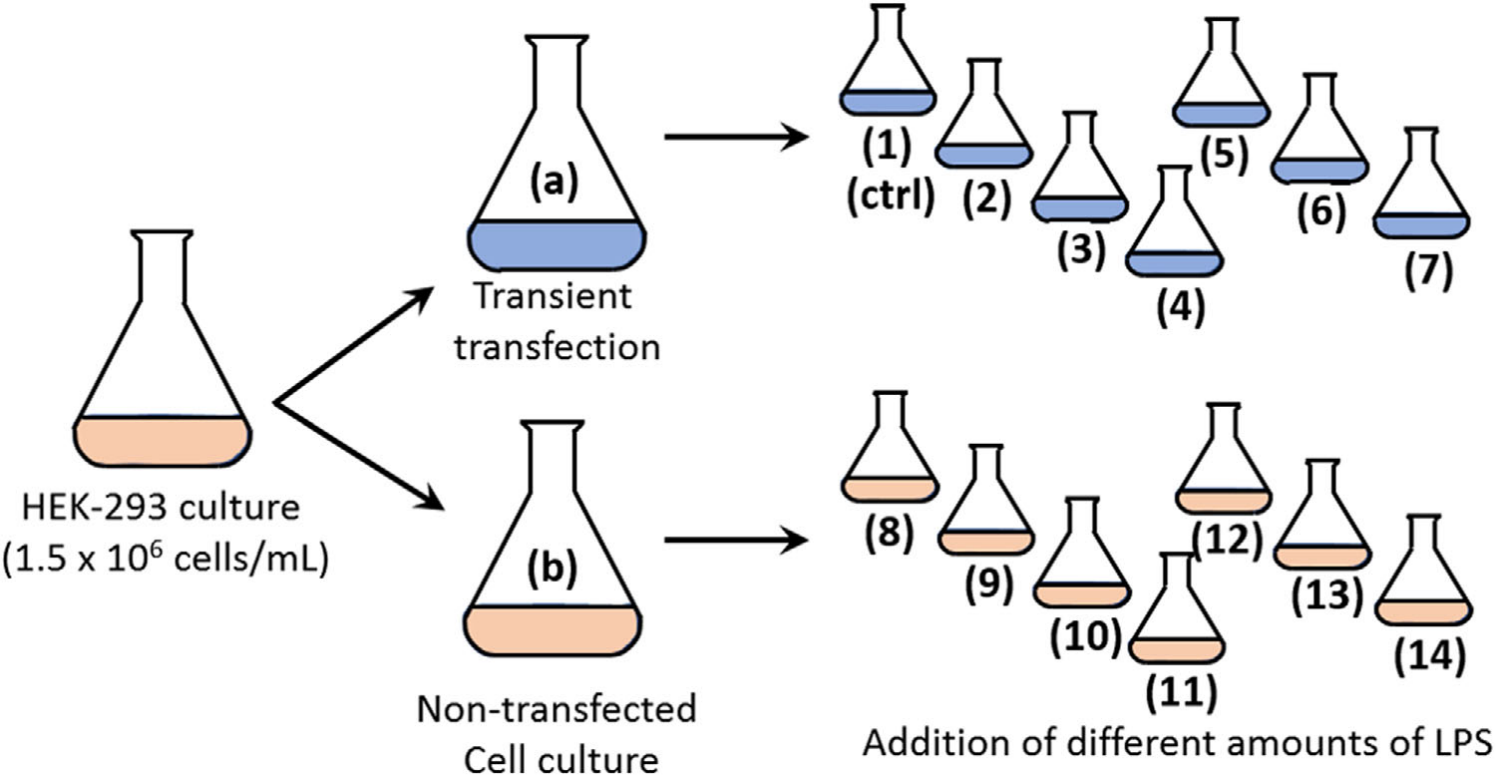
Schematic presentation of the experimental setting. The numbering of the cultures is maintained throughout the study

The plasmids coding for the light and heavy chain for adalimumab were used in a fixed 1:1 stoichiometry for transfection. A schematic overview of the experimental setup is given in Figure 2. The original cell culture was divided in two parts. One part was transfected with plasmids coding for adalimumab, the other part remained non‐transfected. After transfection, aliquots were withdrawn from both cultures for LPS‐detection. Both sub‐cultures were further split and different amounts of LPS were added to the cultures. Immediately after addition of LPS, an aliquot from each culture was again withdrawn and checked for LPS content. The obtained LPS values are given in Table 1. We are aware of the deviations of the calculated versus the measured EU values. There may be many reasons for this. At higher concentrations LPS forms micelles and LPS may interact with cells and medium components. In addition, LPS might also interact with remaining transfection reagent in the culture medium. It was not our intension to look further into LPS distribution in the culture media. This and the fact that cell transfection and cultivation is a complex process was the reason for separate evaluation of the three different experiments and not performing a mean value calculation.

**TABLE 1:** Endotoxin values in subcultures. Culture numeration refers to Figure 2

Culture	Calculated [EU/mL]	Experiment 1 measured [EU/mL]	Experiment 2 measured [EU/mL]	Experiment 3 measured [EU/mL]
(a)	0	<0.5	<0.5	<0.5
(b)	0	<0.5	<0.5	<0.5
(1)	0	<0.5	<0.5	<0.5
(2)	5	1	7	20
(3)	50	10	67	144
(4)	500	48	299	1493
(5)	5000	471	2225	6993
(6)	50,000	4,571	32,147	113,915
(7)	500,000	796,418	391,349	730,469
(8)	0	<0.5	<0.5	1
(9)	5	8	11	40
(10)	50	58	137	280
(11)	500	248	513	2007
(12)	5000	1489	3492	27,692
(13)	50,000	5354	41,020	122,231
(14)	500,000	604,204	383,919	556,322

*Note*: Content of endotoxin is given in EU/mL.

**FIGURE 3:**
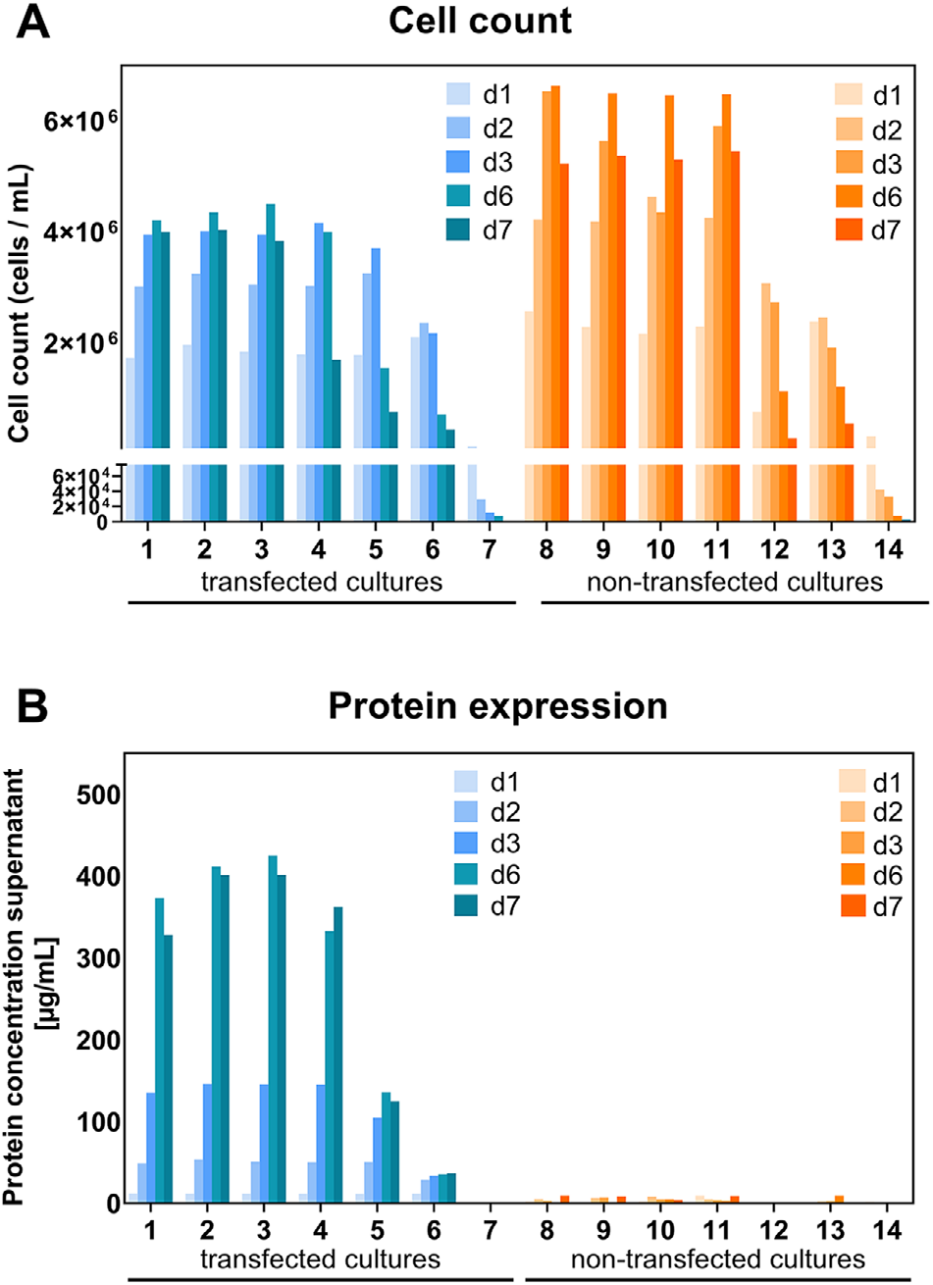
Cell count (A) and antibody titers (B) for experiment 1. First time point is the day after cell culture splitting (d1). Antibody titers were measured directly in the culture supernatants

Cells were cultured for 7 days without being fed further, and, at the indicated time points, samples were withdrawn for cell analyses and antibody concentration determination in the supernatant. The cell counts and antibody titers in the culture supernatant for experiment 1 is shown in Figure 3 and for experiments 2 and 3 in Figures S1 and S2. The non‐transfected cultures have higher cell densities than the transfected cell cultures, albeit this is not so pronounced in experiment 3 (Figure S2). This is not surprising since the transfected cells use energy and nutrients for secreting proteins in the supernatant instead of using these resources for cell growth. The highest cell densities are reached after ∼6 days of cultivation. LPS levels up to (calculated) 500 EU/mL had only marginal impact on cell density (cultures [1–4, 8–11]). Higher LPS concentrations were detrimental for cell growth and in the cultures with highest LPS contents (cultures [7, 14]) cells were readily killed by LPS (Figures 3A, S1A, S2A). Concomitant with cell density the highest antibody concentration in the supernatant were detected after ∼6 days of cultivation (Figures 3B, S1B, S2B). In the untransfected cultures only unspecific binding with values close to the baseline were detected. For transfected cultures without addition of LPS (1), antibody titer after 6 days of expression were approx. 370, 513 and 247 μg/mmL for experiments 1, 2 and 3, respectively. This is within the range that is usually obtained with adalimumab. Interestingly, in experiment 1 the highest antibody concentrations were measured in the supernatants of cultures (2) and (3). Similar, but not so pronounced, the highest antibody concentration in experiment 2 was measured in culture (3). In these cultures, low amount of LPS were present. Antibody titers up to 420 μg/mL were detected in culture (3) (experiment 1) after 6 days of expression. This is an increase of approx. 10% compared to the control cell culture.

Besides cell counting, cell size and percentage of aggregated cells were also determined (Figures S3 and S4). Cells of freshly inoculated cultures had a diameter of ∼16.5 μm, which dropped to ∼12‐ 13 μm after 6 days of cultivation. There was no obvious difference between transfected and non‐transfected cells. This is in accordance with data from a comprehensive metabolic comparative analysis of producing HEK‐293 cells versus the parental HEK‐293 cell line. For both cell lines a cell diameter of 15.5 μm was determined [16]. Cell aggregation was also analysed during cell counting. Especially for cell cultures with high amount of added LPS, accurate cell analysis was not always possible due to increasing rates of cell lysis and remaining cell debris in the cultures.

**FIGURE 4:**
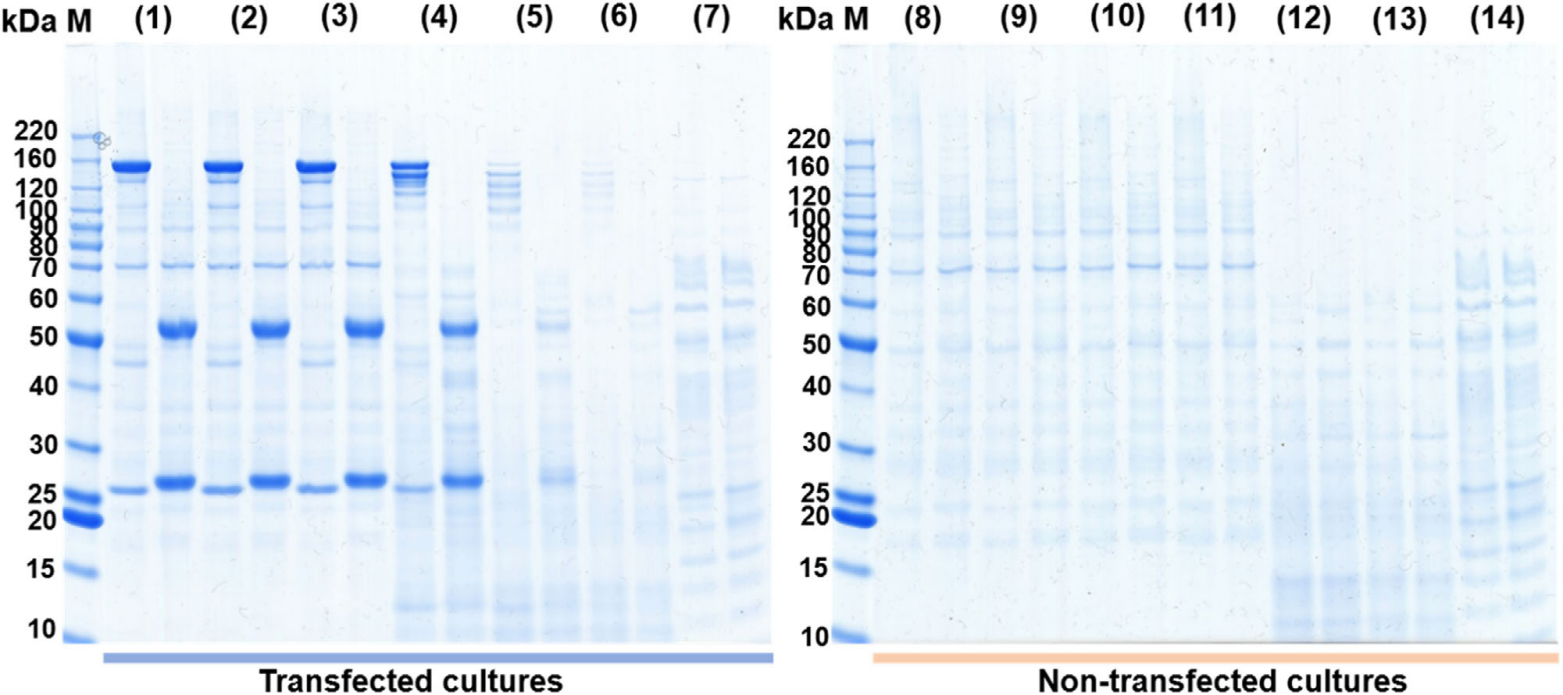
SDS‐PAGE of cell culture supernatants from experiment 1 after 7 days cultivation. Samples were analysed under non‐reducing and reducing conditions (first and second lane of each sample, respectively). The sample numbering refers to the corresponding cell culture as depicted in Figure 1. M: Protein marker

Withdrawn supernatants were also checked by SDS‐PAGE analysis under reducing as well as non‐reducing conditions as shown in Figure 4. Under non‐reducing conditions, a prominent protein band with an apparent molecular weight of ∼160 kDa is visible in cultures (1)–(4). Upon reduction, two bands with apparent molecular weights of ∼55 kDa and 27 kDa, corresponding to the antibody heavy and light chain, can be detected. In culture (4), cells are already compromised by LPS and probably cells are lysed. Upon cell lysis, intracellular proteases are released into the culture, leading to degradation of the antibody as seen as additional bands below the main antibody band appeared. This effect is even more pronounced in cultures (5) and (6). Here, additional proteins released from lysed cells are clearly visible. Interestingly, the protein background pattern for the cultures with the highest LPS levels differ somehow from the pattern observed by the other cultures but are similar to each other.

## 4 DISCUSSION

Working with cell cultures in general requires avoiding any possible contamination. Especially cell cultures from blood‐derived cells are extremely sensitive to the presence of LPS. Concentrations in the ng/mL range of LPS are sufficient to elucidate specific responses. For example, the expression of the anti‐inflammatory cytokine IL10 in mouse macrophages can be induced with concentrations of 10 ng/mL LPS [17]. For the human monocytic leukaemia cell line THP1 cytokine induction is achieved with 5 ng/mL LPS [10]. Non‐blood derived cell lines used frequently in research for transfection studies, such as Cos7, HEK‐293, CHO, or Hela are not significantly compromised regarding cell proliferation and transfection efficiency in the presence of up to 10,000 EU/mL LPS. These LPS levels are much higher than levels that are obtained from standard *E. coli* plasmid preparations [18]. These findings are in accordance with reports that partially purified plasmid DNA can be used for transfecting HEK‐293 cells without compromising protein expression [19, 20]. However, as the experimental setups differ considerably, a head to head comparison of the published data is difficult. Our results suggest that endotoxin‐levels of only up to 500 EU/mL (calculated value) in either transiently transfected or non‐transfected HEK‐293 cell cultures can be tolerated without major distinctive features. At higher LPS concentrations (5000 and 50,000 EU/mL; calculated values) cell proliferation is compromised in transiently transfected as well as in non‐transfected cultures. However, the impact of 5000 EU/mL LPS on the cell count for the transiently transfected culture is not so dramatic as for the non‐transfected culture compared to the lower LPS‐concentrations. The most striking result in our study was that in some cultures with low amount of added LPS (5–50 EU/mL calculated) protein expression could even be increased. Upon high level of recombinant protein expression a situation can occur, when cells cannot cope with folding and assembly of the recombinant protein. The reaction of the cells is the so‐called unfolded protein response (UPR). The UPR leads to enhanced production of chaperones in the endoplasmatic reticulum (ER) which are involved in protein folding [21, 22]. The overexpression or co‐expression of chaperones is nowadays widely used as an approach to enhance recombinant protein expression [23, 24]. Alternative strategies to enhance recombinant protein expression is the addition of small molecule additives to the culture media, for example, dimethyl sulfoxide [25, 26, 27, 28]. The precise mode of action of these small molecule additives is unclear, but at least in mouse embryonic cells it was shown that DMSO can induce ER stress [29]. In earlier studies it was demonstrated that B‐lymphoma cells can be stimulated with LPS to produce high amounts of secreted IgM [30]. It would not be surprising if the upregulation of IgM production is concomitant with overexpression of chaperones. LPS induced ER stress has been reported [31, 32, 33].

In order to specifically respond to the presence of molecules, cells need appropriate receptors. For mammalian cells, the receptors that perceive the presence of LPS belong to the Toll‐like receptor (TLR)‐family. Within this family TLR4 is probably the best characterized receptor. In the human body, LPS can be bound by the plasma protein LPS‐binding protein (LBS). Together with CD14, an integral membrane protein, LPS is transferred to TLR4 and its coreceptor MD2. However, LPS can also directly bind to the TLR4/MD2 complex [14, 15]. Upon activation, TLR4 binds on the intracellular site to the adaptor proteins TIRAP and MyD88 which on their part bind to the interleukin‐1 receptor associated kinase 1 (IRAK1) triggering further downstream signalling [11].

Actually, there are six different HEK cell lines prevalently used for protein expression. Their genomes as well as their transcriptomes have been analysed in a comparative study [34]. According to the transcriptomic data reported in this study, no gene expression data for the main LPS receptor TLR4 in the FreeStyle 293 cell line was detected. However, transcriptomic data for other proteins involved in LPS signalling for example, LBP, MyD88, TIRAP, CD14, IRAK1, TRAF6 or TICAM 1 were clearly detected, albeit at low level. In contrast to TLR4, TLR1 gene expression was detected in all analysed HEK‐293 cell lines. TLR1 is activated by binding to diacylated and triacetylated lipopeptides ([34], extracted and summarized data are given in Table S1).

Since many of the proteins involved in LPS signalling are expressed, we assume that HEK‐293 cells are still able to somehow respond to the presence of LPS. It would be interesting to see, whether the presence of LPS in the culture medium has an impact on gene expression for the proteins in the LPS signalling pathway. But such analyses are far out of the scope of our study. We hypothesize that low LPS concentrations induce ER stress concomitant with upregulation of chaperone expression. This in turn facilitates production of the recombinant protein. However, it remains to be questioned whether addition of LPS to enhance expression levels into a cell culture is advisable.

## CONFLICT OF INTERESTS

The authors are all employees of Sanofi and may hold company shares and/or stock options. The authors declare no additional conflict of interest.

## Supporting information

Supporting Information

## Data Availability

The data that support the findings of this study are available from the corresponding author upon reasonable request.
