# Supplementary material for: Impact of lipopolysaccharides on cultivation and recombinant protein expression in human embryonal kidney (HEK‐293) cells
Source: Eng Life Sci. 2021 Oct 25;21(11):778–85. doi: 10.1002/elsc.202100065 (PMC8576075; doi:10.1002/elsc.202100065)

Supplementary Information

Impact of lipopolysaccharides on cultivation and recombinant protein expression in human embryonal kidney (HEK293) cells

Christine Faust, Christian Beil, Ercole Rao, Thomas Langer*

Sanofi-Aventis Deutschland GmbH, R&D Biologics Research, Building H811, Industriepark Höchst, 65926 Frankfurt am Main

*Corresponding author:

Thomas Langer (thomas.langer@sanofi.com)

Table S1. Transcriptomic data for gene products involved in LPS induced signaling. The transcriptomic data (given as transcripts per kilobase millions, TPM) for the original HEK cell line compared to the Freestyle 293F cell line (Gibco). The original HEK293 cell line is an adherent cell line whereas the FreeStyle 293 F cell line has been adapted to suspension growth in Freestyle 293 medium. In addition to proteins involved in LPS induced signaling transciptomic data two housekeeping proteins, Hsp90 and GAPDH, were extracted as well for comparison reasons.

					**Transcriptomic data (TPM values) (Malm et al. 2020)**					
**Proteins involved in TLR2/4 mediated signaling (Ciesielska et al., 2020; Zamyatina and Heine, 2020; Lannoy et al., 2021)**					**Original HEK293**			**FreeStyle 293-F**		
	**HGNC name** (https://www.genenames.org)	**UniProt ID**	**Function and interaction partner** (https://www.uniprot.org)	**ENSEMBL Gene ID** (http://www.ensembl.org)	(1)	(2)	(3)	(1)	(2)	(3)
LBP	lipopolysaccharide binding protein	P18428	Binds to the lipid A moiety of bacterial lipopolysaccharides (LPS). Acts as an affinity enhancer for CD14.	ENSG00000129988	0,120979	0,0817696	0,221325	0,0884682	0,310747	0,127904
CD14	CD14 molecule	P08571	Coreceptor for bacterial lipopolysaccharide. In concert with LBP binds to monomeric LPS and delivers it to the TLR4/MD2 complex.	ENSG00000170458	2,572599	0,64781894	1,268851	0,0924843	0,113852	0,553214
MD2 (LY96)	lymphocyte antigen 96	Q9Y6Y9	Binds bacterial lipopolysaccharide (LPS). Cooperates with TLR2 and TLR4.	ENSG00000154589	0,186414	0,18295	0	0,204613	0	0
MYD88	MYD88 innate immune signal transduction adaptor	Q99836	Involved in the Toll-like receptor and IL-1 receptor signaling. Acts via IRAK1, IRAK2, IRF7 and TRAF6.	ENSG00000172936	5,867173	6,48120238	7,469238	3,6214196	3,72571165	3,018348
TIRAP (MAL)	TIR domain containing adaptor protein	P58753	Adapter involved in TLR2 and TLR4 signaling. Acts via IRAK2 and TRAF6.	ENSG00000150455	10,21065	7,63318	9,232315	8,260648	9,846641	9,492699
IRAK1	interleukin 1 receptor associated kinase 1	P51617	Recruited by MYD88 to the receptor-signaling complex upon TLR activation. Association with MYD88 leads to IRAK1 phophorylation by IRAK4 and subsequent autophosphorylation and activation.	ENSG00000184216	197,309941	168,678898	199,075793	139,432152	122,559078	128,108009
IRAK2	Interleukin-1 receptor-associated kinase-like 2	O43187	Binds to the IL-1 type I receptor following IL-1 engagement.	ENSG00000134070	1,51243	1,33363	1,85116	2,11621	2,99939	2,79266
IRAK3 (IRAK-M)	interleukin 1 receptor associated kinase 3	Q9Y616	Inhibits dissociation of IRAK1 and IRAK4 from the Toll-like receptor signaling complex by either inhibiting the phosphorylation of IRAK1 and IRAK4 or stabilizing the receptor complex.	ENSG00000090376	0,0430162	0,00849972	0,1180103	0,0181713	0,06629376	0,0264974
IRAK4	interleukin 1 receptor associated kinase 4	Q9NWZ3	Involved in Toll-like receptor (TLR) and IL-1R signaling pathways. Is rapidly recruited by MYD88 to the receptor-signaling complex upon TLR activation to form the Myddosome together with IRAK2.	ENSG00000198001	10,032468	6,544035	8,22449791	6,63711875	7,82464692	7,611479
TRAF6	TNF receptor-associated factor 6	Q9Y4K3	E3 ubiquitin ligase. Conjugates proteins such as IKBKG, IRAK1, AKT1, AKT2.	ENSG00000175104	14,66179	10,41034	12,48567	9,03194	7,82369	9,43827
TICAM1 (TRIF)	toll like receptor adaptor molecule 1	Q8IUC6	Adapter used by TLR3, TLR4 (through TICAM2) and TLR5	ENSG00000127666	6,393694	5,91461	10,2914	4,88021	4,72534	5,13877
TICAM2 (TRAM)	toll like receptor adaptor molecule 2	Q86XR7	In TLR4 signaling, physically bridges TLR4 and TICAM1. In TLR2 signaling, physically bridges TLR2 and MYD88	ENSG00000243414	3,77869	3,7609	4,23024	0,23103	0,31033	0
TRAM1	translocation associated membrane protein 1	Q15629	required for the translocation of secretory proteins across the ER membrane	ENSG00000067167	100,102185	91,121329	125,319563	74,689093	66,390675	64,3603992
IRF7	Interferon regulatory factor 7	Q92985	Key transcriptional regulator of type I interferon (IFN)-dependent immune responses. Activated by TLR signaling.	ENSG00000185507	0	1,443596	1,589136	0,239734	0,7997894	0,440245

**Toll like receptors (TLRs) (Fitzgerald and Kagan, 2020)**										
TLR1	toll like receptor 1	Q15399	Recognition of diacylated and triacylated lipopeptides. Acts via MYD88 and TRAF6.	ENSG00000174125	1,622755	0,417409	1,15925177	1,067448	0,9265108	0,574974
TLR2	toll like receptor 2	O60603	Recognition of lipoproteins or lipopeptides. Can also interact with TLR4 and TLR6. Acts via MYD88 and TRAF6.	ENSG00000137462	0,0175636	0	0,0322056	0	0	0
TLR3	toll like receptor 3	O15455	Nucleotid-sensing, double-stranded RNA. Acts via TICAM1	ENSG00000164342	1,3157773	0,61317	0,855644	0,2777421	0,36309671	0,3083542
TLR4	toll like receptor 4	O00206	Coreceptor for bacterial lipopolysaccharide. Can also interact with TLR2 and TLR6. In concert with LBP binds to monomeric LPS and delivers it to the TLR4/MD2 complex.	ENSG00000136869	9,241759	7,51307	4,98941	0	0	0
TLR5	toll like receptor 5	O60602	Recognizes small molecular motifs named pathogen-associated molecular pattern (PAMPs) expressed by pathogens and microbe-associated molecular patterns (MAMPs) usually expressed by resident microbiota. Acts via MYD88 and TICAM1.	ENSG00000187554	1,338978	0,95063869	0,629334	1,705848	1,34724	1,0656909
TLR6	toll like receptor 6	Q9Y2C9	Specifically recognizes diacylated and, to a lesser extent, triacylated lipopeptides. Can also interact with TLR2 and TLR4. Acts via MYD88 and TRAF6.	ENSG00000174130	0,2207263	0,2326777	0,739106	0,69625196	0,795876	0,692911
TLR7	toll like receptor 7	Q9NYK1	Recognition of uridine-containing single strand RNAs (ssRNAs) of viral origin or guanosine analogs. Acts via MYD88.	ENSG00000196664	0,0149905	0,0150712	0,0599785	0	0,0608018	0
TLR8	toll like receptor 8	Q9NR97	Recognition of RNA degradation products specific to microorganisms that are initially processed by RNASET2. Acts via MYD88.	ENSG00000101916	0	0	0	0	0	0
TLR9	toll like receptor 9	Q9NR96	Recognition of molecular patterns specific to microorganisms. Nucleotide-sensing TLR activated by unmethylated cytidine-phosphate-guanosine (CpG) dinucleotides. Acts via MYD88 and TRAF6.	ENSG00000239732	0	0	0	0	0	0
TLR10	toll like receptor 10	Q9BXR5	Response to microbial agents. Acty via MyD88 and TRAF6.	ENSG00000174123	0,08742186	0,06619396	0,02051017	0,14395837	0,06901122	0,02331701
**Proteins involved in intracellular LPS sensing and pyroptosis (Rathinam et al., 2019; Downs et al., 2020)**										
Card11	caspase recruitment domain family member 11	Q9BXL7	Involved in the costimulatory signal essential for T-cell receptor (TCR)-mediated T-cell activation. Its binding to DPP4 induces T-cell proliferation and NF-kappa-B activation in a T-cell receptor/CD3-dependent manner. Activates NF-kappa-B via BCL10 and IKK. Stimulates the phosphorylation of BCL10.	ENSG00000198286	9,025156	5,46368	4,23507	0	0,0129904	0
Casp4	caspase 4	P49662	Inflammatory caspase that acts as an essential effector of NLRP3 inflammasome-dependent CASP1 activation and IL1B and IL18 secretion in response to non-canonical activators, such as UVB radiation, cholera enterotoxin subunit B and cytosolic LPS	ENSG00000196954	18,696727	12,2436812	10,7393753	2,3276877	0,2269681	1,0245291
Casp5	caspase 5	P51878	Thiol protease that acts as a mediator of programmed cell death. Initiates pyroptosis.	ENSG00000137757	0,23007	0	0	0	0	0
GSDMD	gasdermin D	P57764	Precursor of a pore-forming protein that plays a key role in host defense against pathogen infection and danger signals	ENSG00000104518	3,667252	5,2456427	9,027546	0,0416763	0,110098	0,1676566
**Housekeeping proteins (Picard, 2002; Sikand et al., 2012)**										
Hsp90aa1	Heat shock protein HSP90 alpha	P07900	Molecular chaperone that promotes the maturation, structural maintenance and proper regulation of specific target proteins involved for instance in cell cycle control and signal transduction.	ENSG00000080824	2732,98118	2346,11187	2766,62583	3671,10026	3275,12525	3545,75817
GAPDH	Glyceraldehyde-3-phosphate dehydrogenase	P04406	Has glyceraldehyde-3-phosphate dehydrogenase activity, required for glycolysis.	ENSG00000111640	653,67797	1001,11686	648,015965	1237,90331	1174,24443	1272,01032

References

Ciesielska A, Matyjek M, Kwiatkowska K. TLR4 and CD14 trafficking and its influence on LPS-induced pro-inflammatory signaling [published online ahead of print, 2020 Oct 15]. Cell Mol Life Sci. 2020;10.1007/s00018-020-03656-y. doi:10.1007/s00018-020-03656-y

Downs KP, Nguyen H, Dorfleutner A, Stehlik C. An overview of the non-canonical inflammasome. Mol Aspects Med. 2020;76:100924. doi:10.1016/j.mam.2020.100924

Fitzgerald KA, Kagan JC. Toll-like Receptors and the Control of Immunity. Cell. 2020;180(6):1044-1066. doi:10.1016/j.cell.2020.02.041

Lannoy V, Côté-Biron A, Asselin C, Rivard N. Phosphatases in toll-like receptors signaling: the unfairly-forgotten. Cell Commun Signal. 2021;19(1):10. Published 2021 Jan 25. doi:10.1186/s12964-020-00693-9

Malm M, Saghaleyni R, Lundqvist M, et al. Evolution from adherent to suspension: systems biology of HEK293 cell line development. Sci Rep. 2020;10(1):18996. Published 2020 Nov 4. doi:10.1038/s41598-020-76137-8

Picard D. Heat-shock protein 90, a chaperone for folding and regulation. Cell Mol Life Sci. 2002;59(10):1640-1648. doi:10.1007/pl00012491

Rathinam VAK, Zhao Y, Shao F. Innate immunity to intracellular LPS. Nat Immunol. 2019;20(5):527-533. doi:10.1038/s41590-019-0368-3

Sikand K, Singh J, Ebron JS, Shukla GC. Housekeeping gene selection advisory: glyceraldehyde-3-phosphate dehydrogenase (GAPDH) and β-actin are targets of miR-644a. PLoS One. 2012;7(10):e47510. doi:10.1371/journal.pone.0047510

Zamyatina A, Heine H. Lipopolysaccharide Recognition in the Crossroads of TLR4 and Caspase-4/11 Mediated Inflammatory Pathways. Front Immunol. 2020;11:585146. Published 2020 Nov 27. doi:10.3389/fimmu.2020.585146

Figure S1. Cell count (A) and antibody titers (B) for experiment 2. First time point is the day after cell culture splitting (d1). Antibody titers were measured directly in the culture supernatants.

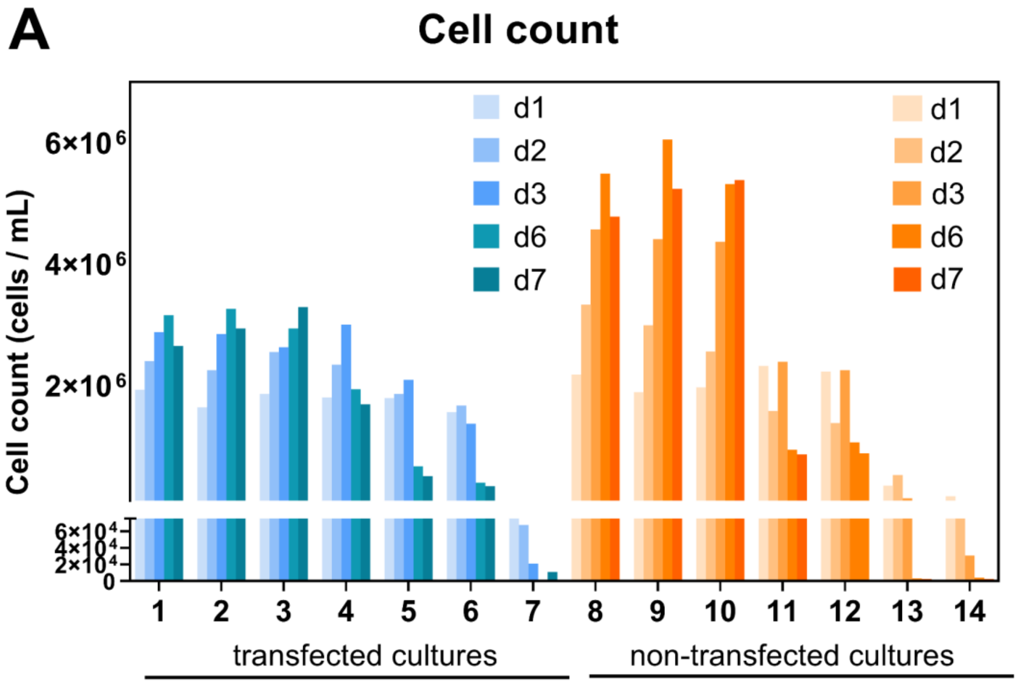

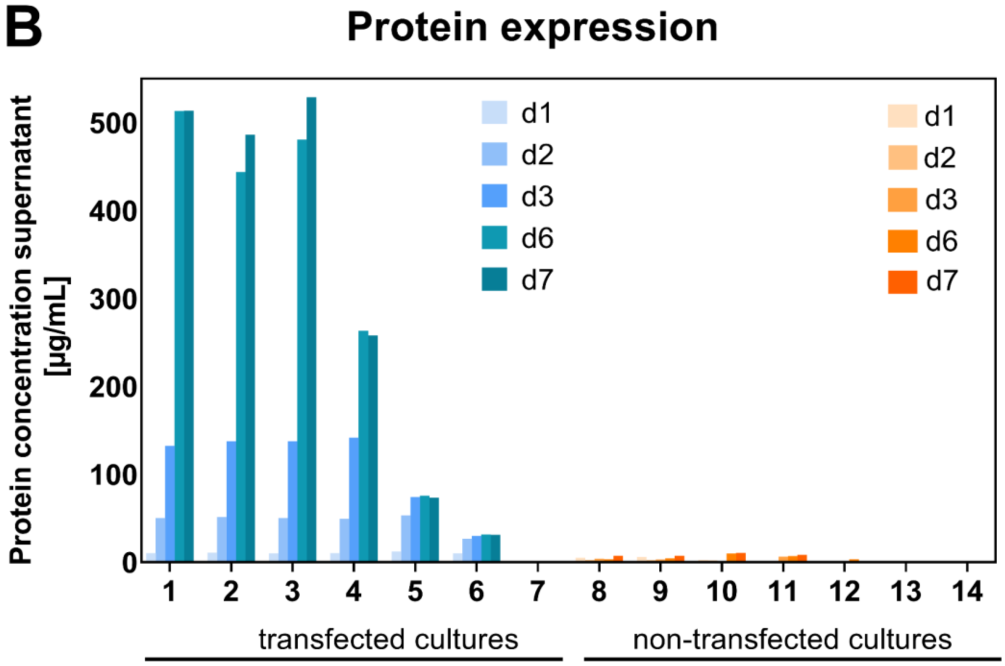

Figure S2. Cell count (A) and antibody titers (B) for experiment 3. First time point is the day after cell culture splitting (d1). Antibody titers were measured directly in the culture supernatants.

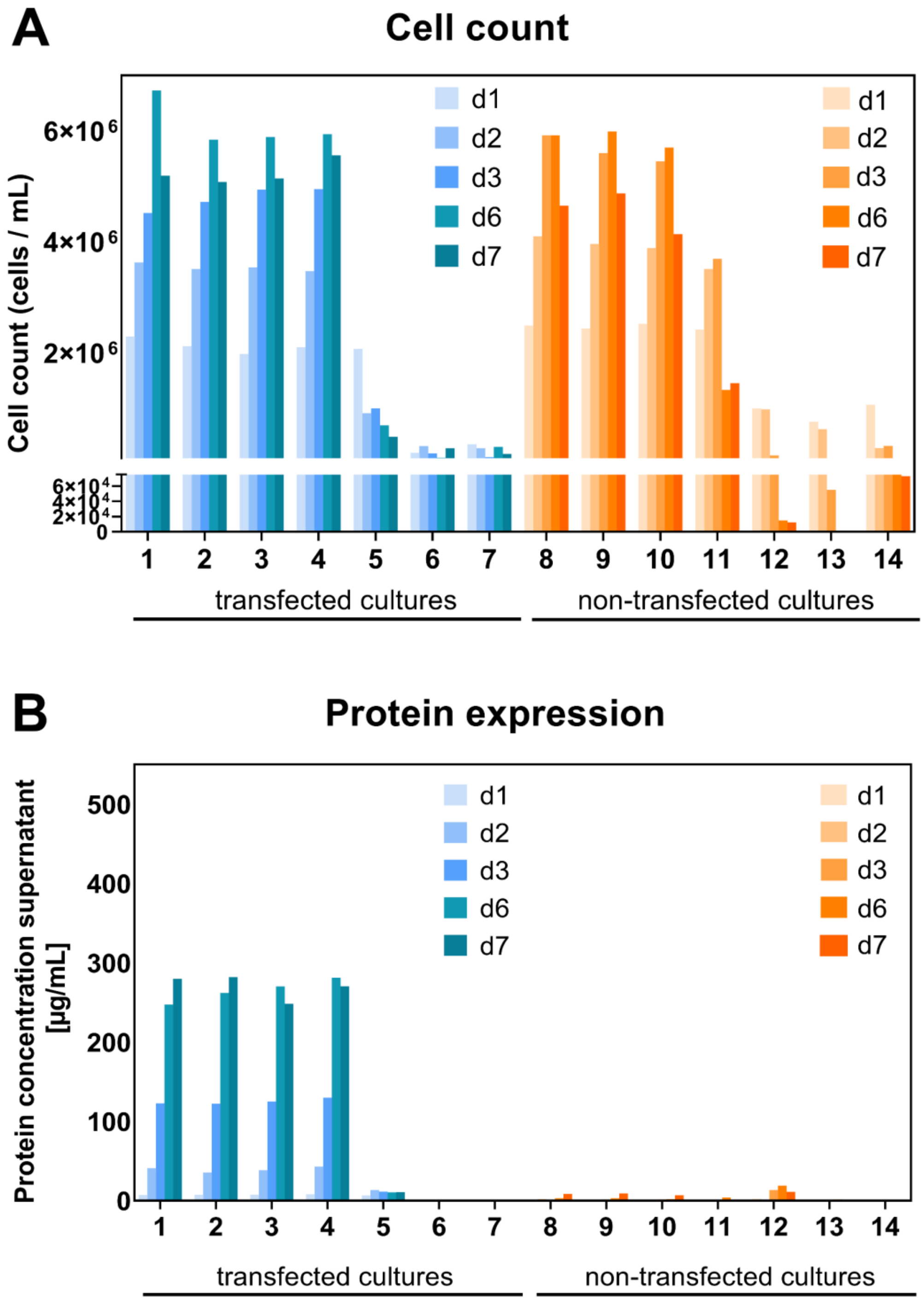

Figure S3. Cell size for the three different and independent experiments (A)-(C).

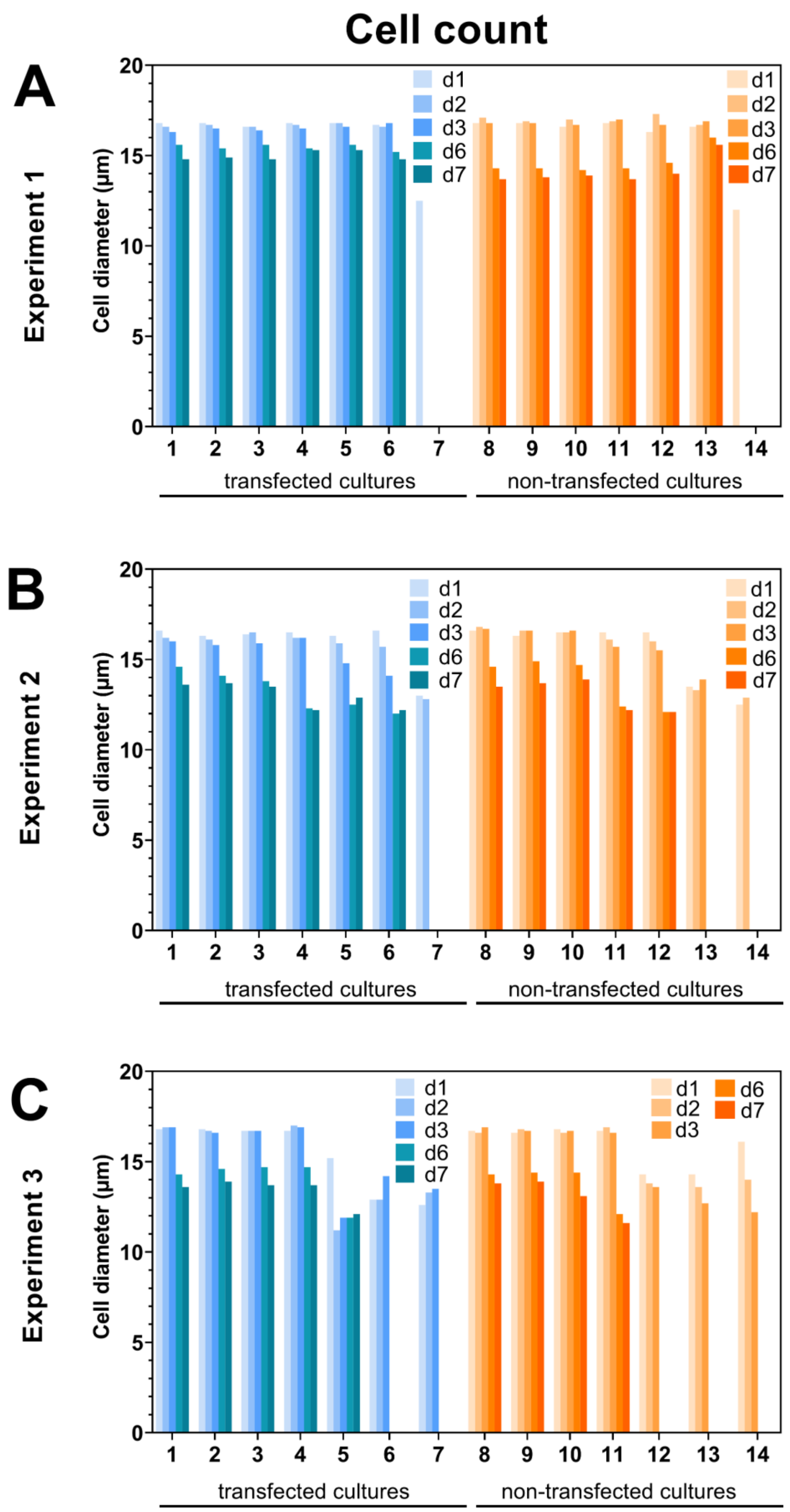

Figure S4. Cell aggregation of the cell cultures for the three different experiments (A)-(C). Cells were classified as "aggregates" when five or more cells stick together as interpreted by the NucleoView NC-200 software. If no value is given a reliable measurement was not possible due to increasing cell clumping and cell lysis.

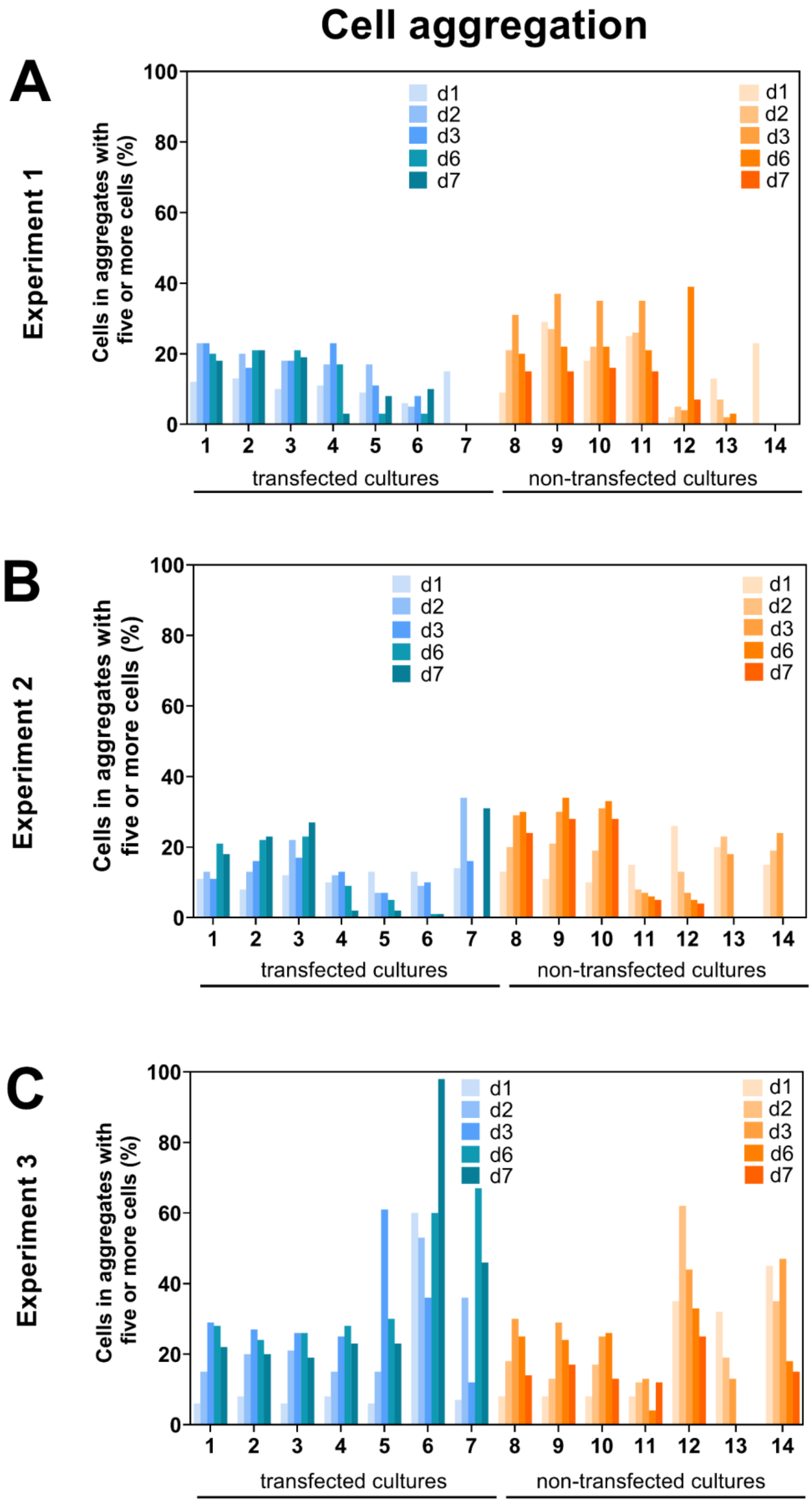

Supplement: Supplementary file 1 — Supporting Information [file ELSC-21-778-s001.pdf]
